# Multivariate versus univariate spectrum analysis of dentine sialophosphoprotein (DSPP) for root resorption prediction: a clinical trial

**DOI:** 10.1186/s12903-022-02178-2

**Published:** 2022-04-29

**Authors:** Mohd Norzaliman Mohd Zain, Zalhan Md Yusof, Katrul Nadia Basri, Farinawati Yazid, Yong Xian Teh, Asma Ashari, Shahrul Hisham Zainal Ariffin, Rohaya Megat Abdul Wahab

**Affiliations:** 1 MIMOS Berhad, Technology Park Malaysia, 57000 Kuala Lumpur, Malaysia; 2 Faculty of Electrical Engineering, Universiti Teknologi MARA, 40450 Shah Alam, Selangor Malaysia; 3 Department of Family Oral Health, Faculty of Dentistry, Universiti Kebangsaan Malaysia, 50300 Kuala Lumpur, Malaysia; 4 Department of Biological Sciences and Biotechnology, Faculty of Science and Technology, Universiti Kebangsaan Malaysia, 43600 Bangi, Malaysia

**Keywords:** Absorption spectroscopy, DSPP, ELISA, Multivariate analysis, Root resorption, Univariate analysis

## Abstract

**Background:**

A force applied during orthodontic treatment induces inflammation to root area and lead to root resorption known as orthodontically induced inflammatory root resorption (OIIRR). Dentine sialophosphoprotein (DSPP) is one of the most abundant non-collagenous proteins in dentine that was released into gingival crevicular fluid (GCF) during OIIRR. The aim of this research is to compare DSPP detection using the univariate and multivariate analysis in predicting classification level of root resorption.

**Methods:**

The subjects for this study consisted of 30 patients in 3 group classified as normal, mild, and severe groups of OIIRR. The GCF samples were taken from upper permanent central incisors in the normal and mild group while the upper primary second molars in the severe group. The DSPP qualitative detection limit was determined by analyzing the whole absorption spectrum utilizing multivariate analysis embedded with different preprocessing method. The multivariate analysis represents the multi-wavelength spectrum while univariate analyzes the absorption of a single wavelength.

**Results:**

The results showed that the multivariate analysis technique using partial least square-discriminate analysis (PLS-DA) with the preprocess method has successfully improved in classification prediction for the normal and mild group at 0.88 percent accuracy. The multivariate using PLS-DA algorithm with Mean Center preprocess method was able to predict normal and mild tooth resorption classes better than the univariate analysis. The classification parameters have improved in term of the specificity, precision and accuracy.

**Conclusion:**

Therefore, the multivariate analysis helps to predict an early detection of tooth resorption complimenting the sensitivity of the univariate analysis.

*Trial registration* NCT 05077878 (14/10/2021).

## Background

Orthodontically induced inflammatory root resorption (OIIRR) is one of the most significant iatrogenic damage associated with orthodontic tooth movement and has gained a lot of attention from the clinicians [1]. The prevalence of OIIRR can be up to 91% of orthodontic patients [2], and in most circumstances, it is usually of mild degree and clinically insignificant. However, for susceptible patients, OIIRR may result in severe resorption of more than 4 mm or one-third of the original root length in 2.9–6.25% of teeth [3, 4] which may jeopardize the prognosis of orthodontic treatment [5]. Currently, the most widely used method to monitor root resorption clinically is through periodic radiographic method. The conventional 2-D radiographs such as a periapical film or panoramic film are readily available at almost all dental clinics and easy to use, however they have the disadvantages of difficulties in standardisation, overlapping structures and magnification issues. The 3D imaging such as cone beam computed tomography (CBCT) can eliminate the shortcomings mentioned, however, it is not routinely taken in all orthodontic patients due to its costs and high radiation dose. Moreover, early detection of OIIRR is not possible as the radiographs only show root shortening when 60–70% of the mineralised tissue has already been lost [6]. Thus, many researchers have attempted to develop a safer and more sensitive alternative such as the biological marker method in diagnosing OIIRR. Previous research works have proved that protein biomarker can monitor the root resorption during orthodontic tooth movement. Dentine sialophosphoprotein (DSPP) is the major non-collagenous protein in the dentine [7] and was recommended as a potential biomarker of root resorption as it is only released into the surrounding periodontal space as a by-product of dentine breakdown during active root resorption [8, 9]. Gingival crevicular fluid (GCF) is an inflammatory transudate that consists of several cellular factors, tissue breakdown products, inflammatory mediators and antibodies in response to changes in the oral microenvironment [10]. Thus, DSPP from GCF is commonly used as a highly dentine-specific biological marker to quantify the severity of OIIRR [9, 11, 12]. There are a few widely used methods in detecting proteins in GCF such as Enzyme-Linked Immunosorbent Assay (ELISA), Western blotting and enzyme assay. ELISA method is a popular method that uses antibodies and colour change in identifying and quantifying a target protein. It is widely used due to its simplicity, flexibility and the enzymatic signal amplification enabling high sensitivity [13]. In ELISA, the test sample will react with primary and secondary antibodies, resulting in the antigen molecules being sandwiched between the solid phase and enzyme-linked antibodies. In early detection of OIIRR, it is important to have a more sensitive diagnostic testing at a very low level of DSPP volume. However, detection using ELISA lacks of accuracy and specificity in detecting the sample at trace level and depends only on an absorption of a single wavelength. Therefore, the purpose of this study is to prove that multi wavelength analysis of biological markers spectrum can give a better conclusive result [14, 15]. The objective of this study is to investigate the univariate and multivariate analysis of the ELISA spectrum trace samples. Currently, the spectrophotometer determined DSPP level using ELISA by measuring the single wavelength peak at 450 nm. Therefore, only the absorption intensity at that particular wavelength is measured from the ELISA samples. Hence, this single wavelength is using univariate analysis while multivariate analysis represents the whole ELISA sample spectrum compared by measuring the classification performance parameters such as sensitivity, specificity, precision and accuracy. The study will use a qualitative multivariate regression model called PLS-DA, a combination of partial least square (PLS) and discriminate analysis (DA). The spectrum used in the model is introduced with different preprocesses methods such as Autoscale, standard normal variate (SNV) and Mean Center. The multivariate algorithm prediction and validation process will classify the ELISA sample containing DSPP biomarker into the selected classes of normal, mild and severe group.

## Methods

### Aims

The aim of this study is to compare DSPP detection using the univariate and multivariate analysis in predicting classification level of root resorption.

### Subject selection

Ethical approval was obtained from the Research Ethics Committee, Universiti Kebangsaan Malaysia (Ethical approval number, UKM PPI/111/8/JEP-2018-438). Clinical trials in this study has been registered with approval number NCT 05077878 (14/10/2021). Informed consent from the subjects and their parents were obtained prior to the study commencement. All subjects who participated in this study were recruited from the Postgraduate Orthodontic Clinic, Faculty of Dentistry, Universiti Kebangsaan Malaysia. Thirty patients were recruited having met the following inclusion criteria of (1) good general health, (2) good periodontal health, (3) caries free, (4) good oral hygiene. The subjects were divided into 3 groups based on a radiographic assessment on the level of root resorption; normal (no root resorption), mild (less than 2 mm) and severe (more than 2 mm). The GCF samples were taken from the permanent central incisors of untreated subjects in the normal control group (n = 14), while in the mild group, samples were collected from the permanent central incisors with mild root resorption (< 2 mm) in patients undergoing active orthodontic treatment after 6 months with radiographic evidence (mild group, n = 5. Patients with severe root resorption (> 2 mm) were those with primary second molars undergoing physiological root resorption (severe group, n = 11).

### Gingival crevicular fluid (GCF) sampling

Informed consent was obtained from the patients, or the guardians of those under 18 years of age, prior to the study. The GCF samples were taken from the sulcus of upper permanent central incisors in the normal and mild group while in the severe group the samples were acquired from the upper primary second molars. The involved teeth were isolated with cotton rolls together with gently dried of tooth surface using air stream for 5 s before starting GCF sampling and a saliva ejector was used during the procedure for the prevention of salivary contamination. The GCF samples were collected from the mesial and the distal gingival crevicular margins of the involved teeth using gingival fluid collection strips (Periopaper, Oraflow, Smithtown, N.Y.). The paper strips were placed within the sulcus (intrasulcular method) approximately 1–2 mm into the gingival sulcus and left in situ for 60 s [12]. Every sample collection was taken 3 times at the same site with one-minute resting intervals to allow GCF to be replenished into the gingival sulcus. Next, the paper strips were inserted immediately into a 1.5 mL microcentrifuge tube containing 500 μL of protease inhibitor (Cocktail Kit, MP Biomedicals, LLC, US). The paper strip with GCF was eluted by centrifugal filtration at 400 × *g* for 10 min at 4 °C with centrifuge machine (Heraeus Fresco 21 centrifuge, Thermo Scientific) to extract the samples from the paper strips. The samples were stored at − 80 °C before the laboratory analysis.

### Enzyme-linked immunosorbent assay (ELISA) samples characterization

The standard and test samples were made triplicate using Human DSPP ELISA kit specific for each enzyme (Wuhan Fine Biotech Co. Ltd., China). Additionally, the technique was used for detection specific protein for each enzyme present in the samples. The ELISA was carried out essentially when the anti-DSPP antibody was pre-coated onto 96-well plates and the biotin conjugated anti-DSPP antibody was used as detection antibodies. About 100 µL of standards and GCF samples were diluted with the sample dilution buffer respectively and were added to the well subsequently. All the samples were incubated at 37 °C for 90 min. The plate was washed with 350 µL wash buffer to discard the solution. About 100 µL biotin conjugated detection antibody and antibody dilution buffer diluted at 1:100 was added to the well and incubated at 37 °C for 60 min. About 100 µL of horseradish peroxidase (HRP)-Streptavidin was added and unbound conjugates were washed away with wash buffer, incubated for 30 min. Then, 90 µL of tetramethylbenzidine (TMB) substrates were added into each well and incubated in the dark for 15–30 min to visualize HRP enzymatic reaction. HRP catalyzed TMB to produce a blue colour product that changes into yellow after adding the acidic stop solution. The sandwich ELISA used for quantitative detection of DSPP in GCF samples obtained from orthodontic patients with three different groups. Current ELISA analysis was done by evaluating the optical density absorbance at 450 nm of standard and GCF samples in a microplate reader. The spectrophotometer instrument (Thermo Varioskan Flash, Thermo Scientific) utilizes a Xenon flash lamp to produce a very low stray light and a rotating grating in the monochromator for wavelength selection. Therefore, the scanning optics able to provide 1 nm resolution from 200 to 600 nm wavelength for spectrum scanning measurement. The absorbance wavelength of each group (normal, mild and severe) is obtained from ELISA microplate reader to determine its standard curve and the DSPP concentration. The absorbance values at a single wavelength at 450 nm and the absorbance spectrums were recorded for every sample. The data was then analyzed and further discussed in the next section.

## Results

**Fig. 1:**
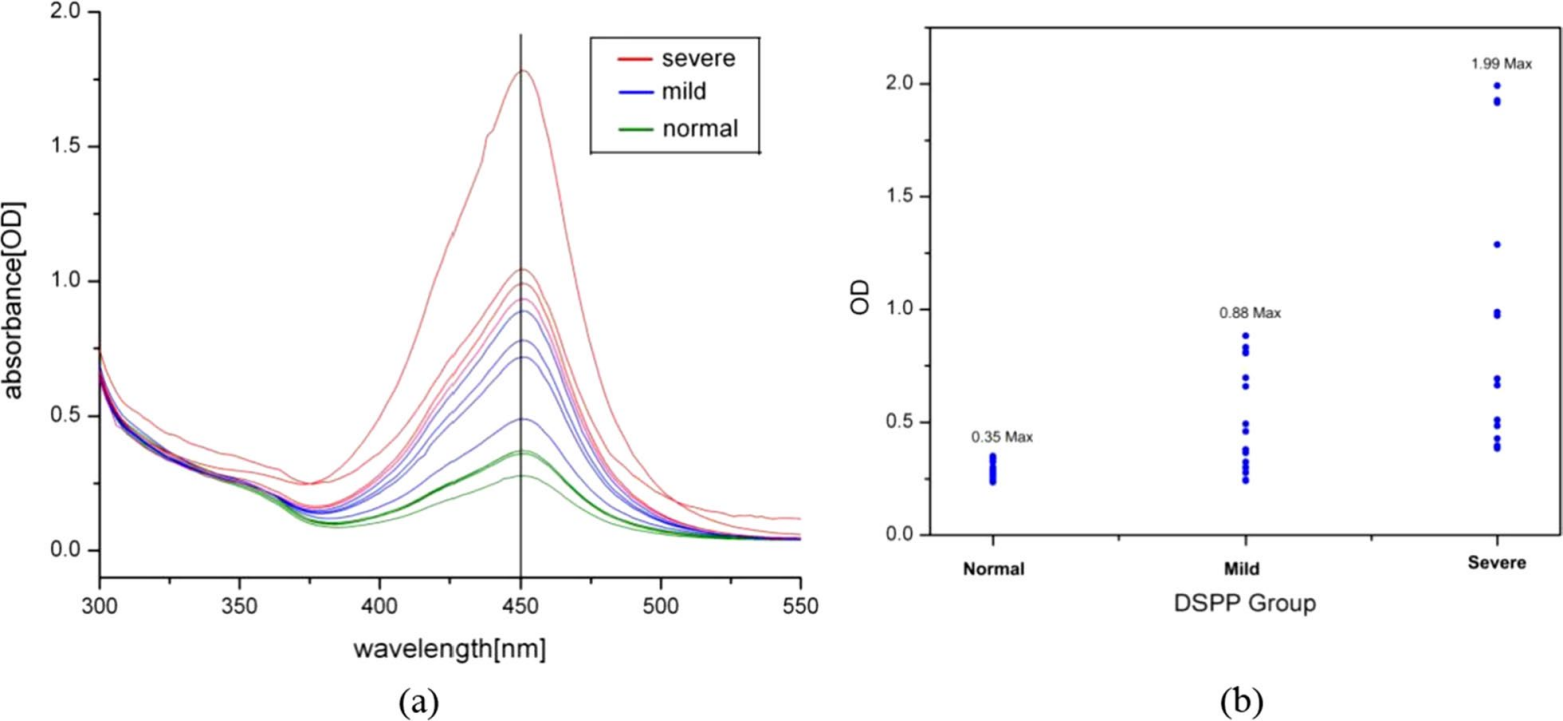
**a** Absorbance spectrum of samples at 450 nm. **b** Bar chart of a translated absorption into quantitative optical density (OD)

The univariate method in determination of DSPP level through the optical density (OD) value or the single wavelength absorption value set at 450 nm. Analysis showed in Fig. 1a where the absorbance spectrum of the 30 ELISA samples were ranged from 300 nm wavelength until 550 nm wavelength. Typically, all ELISA samples showed different absorbance intensity at 450 nm wavelength according to the DSPP level. The DSPP level was categorized into three groups which are the normal, mild and severe group. In the graph, the absorbance intensity was referring to the OD of the samples with the lower intensity absorbance indicated for lower DSPP level while higher absorbance indicated for a higher DSPP level. All OD value of 30 ELISA samples at 450 nm wavelength later translated into a bar chart shown in Fig. 1b. In the bar chart, the maximum OD value of the normal group is the minimum value of the mild group (OD value at 0.35) while the maximum value of the mild group is the minimum OD value of the severe group (OD value at 0.88). The highest OD value for the severe group sample is 1.99 as shown in Fig. 1b. Hence, in this univariate analysis, the OD range value of the actual classes and the predicted classes for each group was determined from the mentioned values. Some of the samples have overlap OD values between the categorized groups, which can be seen either between normal with the mild group or between mild with the severe group. All OD values from the samples then translated into the confusion matrix table of the actual group classes and the predicted classes as depicted in Table 1a. For example, 9 samples have the OD value below than 0.35. This means only 9 samples which have the same actual and the predicted class. In the severe group, only 6 samples have OD values below than 0.88, which means the sample actual class group is severe but predicted as mild. The sensitivity, specificity, precision, and accuracy calculated using the formula and the true table depicted in Table 1b showed overall accuracy was at 0.84 in univariate analysis.

**Table 1:** Translation of OD values from the samples into the confusion matrix table

	Actual classes		
	Normal	Mild	Severe
*(a) The confusion matrix table consists of normal, mild, and severe group*			
Predicted classes			
Normal	9.0	0.0	0.0
Mild	1.0	10.0	6.0
Severe	0.0	0.0	4.0
*(b) True table analysis for each group from all*			
True positive (TP)	9	10	4
False positive (FP)	1	6	0
True negative (tn)	20	13	20
False negative (FN)	0	1	6

Parameter	Value			
		Normal	Mild	Severe
*(c) Parameters formula and its calculated value of sensitivity, specificity, precision, and model accuracy*				
$\text{Sensitivity} = \frac{\text{TP}}{\text{TP+FN}}$	(i)	1.00	0.91	0.40
$\text{Specificity} = \frac{\text{TN}}{\text{TN+FP}}$	(ii)	0.95	0.68	1.00
$\text{Precision} = \frac{\text{TP}}{\text{TP+FP}}$	(iii)	0.90	0.62	1.00
$\text{Accuracy} = \frac{\text{TP+TN}}{\text{TP+TN+FP+FN}}$	(iv)	0.96	0.76	0.80

A confusion matrix in Table 2 showed the analysis result of sample groups (normal, mild and severe) tabulated into calibration and validation domain, for each preprocesses method. A confusion matrix with no preprocess method introduced was also shown in Table 2a as a reference. Calculated performance parameters using the same formula in the univariate analysis in Table 1c for the sensitivity, specificity, and the precision. Table 3 showed the result of the performance classification parameter which includes the sensitivity, the specificity and the precision using PLS-DA multivariate analysis model. The comparison of the result analysis of the four multivariate models with no preprocesses or different preprocesses method were also done. Multivariate analysis without implementing preprocesses method showed high specificity result for all normal, mild, and severe group. The specificity formula is shown in Table 1c where high specificity means lower false positive (FP) value for that specific class. For example, the specificity for the normal group depicted in Table 3a was 93 percent. The value was for the normal group as a specific class alone and the specificity calculated by using the multivariate algorithm with no preprocess’s method. The number of calculated 126 variables acquired from near infra red (NIR) spectrum wavelength with one FP sample whereas one mild group sample was identified as normal group as shown in Table 2a. However, the specificity value increased to 100 percent shown in Table 3d after being analyzed with the multivariate algorithm embedded with preprocessing mean center method. It is indicated that the variables treated with preprocessing mean center method successfully increased the specificity value. The validation result also exhibits the same performance as the calibration domain. In term of sensitivity, the multivariate algorithm with preprocessing auto scale gave higher sensitivity for normal and mild class shown in Table 3b compared to multivariate algorithm embedded with preprocessing mean center which gives higher sensitivity in the normal and severe group class. Therefore, PLS-DA with the auto scale method was more suitable for early detection of root resorption. Nevertheless, PLS-DA coupled Mean Center depicted in Table 3d indicated higher precision and accuracy compared to multivariate with other preprocesses methods including with no preprocess method applied. In the actual scenario, there were a few mild samples in the mild group identified as normal group and some samples in the severe group identified as mild group. The multivariate algorithm helps to make an exact prediction for these samples. The prediction performance is measured by the classification performance parameters such as the specificity, precision and accuracy. The PLS-DA with SNV had increased the specificity to 71 percent from 68 percent for the mild group using univariate analysis. The specificity in the normal group is 93 percent while PLS-DA with SNV and PLS-DA with Mean Scale respectively is 100 percent compared to 95 percent using univariate analysis. The higher precision value observed for the normal and mild group at 100 percent and 75 percent using multivariate, PLS-DA with Mean Scale better than the precision value of 90 percent and 62 percent calculated using univariate shown in Tables 1a and 3d. Finally, the accuracy slightly increased in the normal group from 96 percent using the univariate analysis to 100 percent utilizing multivariate algorithm, PLS-DA with Mean Center. Nevertheless, the accuracy value for the mild group remains the same for both univariate and multivariate, PLS-DA with Mean Center model.

**Table 2:** The confusion matrix table of number sample for each group

	Calibration			Validation		
	Actual classes			Actual classes		
	Normal	Mild	Severe	Normal	Mild	Severe
(a) Spectral data with no preprocess						
*Predicted classes*						
Normal	7	1	0	3	0	0
Mild	0	2	1	0	2	0
Severe	0	4	6	0	1	3
(b) Spectral data with autoscale						
*Predicted classes*						
Normal	7	0	0	3	0	0
Mild	0	5	5	0	3	0
Severe	0	2	2	0	0	3
(c). Spectral data with SNV						
*Predicted classes*						
Normal	6	0	1	3	0	3
Mild	0	4	4	0	3	0
Severe	1	3	2	0	0	0
(d) Spectral data with mean center						
*Predicted classes*						
Normal	7	0	0	3	0	0
Mild	0	3	1	0	2	0
Severe	0	4	6	0	1	3

**Table 3:** The performance measure of sensitivity, specificity and precision based on the PLS-DA model embedded with (a) no preprocesses method (b) with auto scale (c) with SNV and (d) with mean center

Parameter	Calibration			Validation		
	Normal	Mild	Severe	Normal	Mild	Severe
*(a) PLS-DA with no preprocesses*						
Sensitivity	1.00	0.29	0.86	1.00	0.67	1.00
Specificity	0.93	0.93	0.71	1.00	1.00	0.83
Precision	0.88	0.67	0.60	1.00	1.00	0.75
Accuracy	0.95	0.71	0.76	0.43	0.38	0.38
*(b) PLS-DA with autoscale*						
Sensitivity	1.00	0.71	0.29	1.00	1.00	1.00
Specificity	1.00	0.64	0.86	1.00	1.00	1.00
Precision	1.00	0.50	0.50	1.00	1.00	1.00
Accuracy	1.00	0.67	0.67	1.00	1.00	1.00
*(c) PLS-DA with SNV*						
Sensitivity	0.86	0.57	0.29	1.00	1.00	0.00
Specificity	0.93	0.71	0.71	0.50	1.00	1.00
Precision	0.86	0.50	0.33	0.50	1.00	1.00
Accuracy	0.90	0.67	0.57	0.67	1.00	0.67
*(d) PLS-DA with mean center*						
Sensitivity	1.00	0.43	0.86	1.00	0.67	1.00
Specificity	1.00	0.93	0.71	1.00	1.00	0.83
Precision	1.00	0.75	0.60	1.00	1.00	0.75
Accuracy	1.00	0.76	0.76	1.00	0.89	0.89

## Discussions

In our study, the GCF sample was collected from sulcus of the maxillary central incisors of patients according to the selection criteria. The maxillary central incisor was selected as it is the most accessible tooth for collection of GCF. It also has the highest percentage of severe root resorption due to orthodontic tooth movement [5]. The subjects selected were excluded from having any history of dental trauma as dental trauma may induce inflammation and root resorption [16]. The duration of force application or active treatment is also one of the risk factors related to orthodontic treatment as well as increased levels of apical root resorption [5], causing a smaller number of suitable patients for mild group in this study within 6-month of orthodontic treatment. The ELISA method utilizes the spectroscopic technique of one specific wavelength.

Spectroscopic measurements are very sensitive and non-destructive and require only very small amounts of material (GCF sample) for analysis [17, 18] which number of sample size of patients should not be critical especially in patients undergoing orthodontic treatment. The absorption intensity of the sample for a single wavelength is calculated accordance to Lambert Law,

1 $$- \log \;{\text{T}} =\upvarepsilon {\text{bc}},$$

where the transmittance, T is the ratio of light intensity passed through the sample, after and before. The transmittance is equalled to the product of the molar absorptivity of target protein (e), the light path of sample (b), and the concentration of the compound in the solution (c). Since the molar absorptivity of the target protein and the light path of the measured sample in the microplate well remain unchanged, therefore the absorption of the DSPP in the GCF sample depends linearly on its concentration. In the univariate method, determination of DSPP level is through the OD value or the single absorption wavelength value set at 450 nm.

The sensitivity, specificity, precision, and accuracy were calculated using the formula and the true table. It is important to note that the calculation in the true table is for the specific group. If calculation is specific for normal group, correctly detecting normal samples as normal is known as true positive (TP) case and the correctly detecting non-normal samples as non-normal are known as true negative (TN). While false negative (FN) is where normal samples are incorrectly identified as non-normal and FP is when non-normal samples are incorrectly identified as normal. The accuracy is by dividing the number of the correctly classified sample with the total number of samples. The correctly classified sample in this study was the sum of the correctly classified sample of the normal, mild and severe group.

In the multivariate analysis, multiple wavelengths from 300 nm until 550 nm was used instead of single wavelength at 450 nm for univariate analysis. The multiple wavelengths represent the variables of the multivariate analysis from the spectral acquired in the experiment. At first, a preprocessing method was applied to the variables beforehand to reduce the noise. A few different preprocess methods such as SNV, mean center and autoscale were used in this analysis. Then, the preprocessed data were analyzed qualitatively using PLS-DA, a combination of PLS and a classification algorithm, DA. PLS-DA is an algorithm used for predictive modelling as well as for discriminative variable selection. The total of ELISA samples was split into calibration and validation domain at 7:3 ratio because of the low total samples collected. The multivariate algorithm helps to make an exact prediction for these samples. The prediction performance is measured by the classification performance parameters such as the specificity, precision and accuracy. The multivariate algorithm coupled with SNV or Mean Center preprocesses have increased the specificity of the measured samples more than using univariate analysis [12].

DSPP trace level using ELISA is aimed for early detection of tooth resorption during orthodontic treatment. Hence, the analysis was concentrated at normal and mild classes. The univariate analysis gives the highest sensitivity value than the multivariate analysis in the normal and mild group. It is justified that univariate is represent by a single wavelength. The single wavelength observed at 450 nm has the most absorption intensity shown in the absorbance spectrum of the ELISA sample. However, multivariate analysis improves the classification processes in narrowing the gap specificity between the group classes and overlap between classes. The multivariate analysis using PLS-DA algorithm with Mean Center preprocess method successfully improves the DSPP trace which reflected in its increased specificity, precision and accuracy values for the normal and mild group.

## Conclusions

The multivariate algorithm has proven to be able to predict normal and mild tooth resorption at 0.88. In average, the accuracies value is better than univariate analysis. The accuracy may be increased if more high-quality samples are analyzed and collected from the orthodontic patients.

## Data Availability

The datasets generated and/or analyzed during the current study are not publicly available as it requires approval from the sponsor but are available from the corresponding author on reasonable request.

## Abbreviations

OIIRR: Orthodontically induced inflammatory root resorption
DSPP: Dentine sialophosphoprotein
GCF: Gingival crevicular fluid
PLS-DA: Partial least square discriminate analysis
CBCT: Cone beam computed tomography
ELISA: Enzyme-linked immunosorbent assay
PLS: Partial least square
DA: Discriminate analysis
SNV: Standard normal variate
HRP: Horseradish peroxidase
TMB: Tetramethylbenzidine
OD: Optical density
FP: False positive
NIR: Near infra red
TP: True positive
TN: True negative
FN: False negative
